# A putative N-terminal nuclear export sequence is sufficient for Mps1 nuclear exclusion during interphase

**DOI:** 10.1186/s12860-015-0048-6

**Published:** 2015-02-27

**Authors:** Haiwei Jia, Xiaojuan Zhang, Wenjun Wang, Yuanyuan Bai, Youguo Ling, Cheng Cao, Runlin Z Ma, Hui Zhong, Xue Wang, Quanbin Xu

**Affiliations:** Navy General Hospital of China, Beijing, 100048 China; State Key Laboratory for Molecular Developmental Biology, Institute of Genetics and Developmental Biology, Chinese Academy of Sciences, Beijing, 100101 China; Beijing Institute of Biotechnology, Taiping road 27, POB 130(8), Beijing, 100850 China; Department of Life Science, Anhui University, Hefei, 230601 China; West China Hospital, Sichuan University, Chengdu, 610041 China

**Keywords:** Mps1 kinase, Subcellular distribution, Crm1, Nuclear export sequence

## Abstract

**Background:**

Mps1, an essential component of the mitotic checkpoint, is also an important interphase regulator and has roles in DNA damage response, cytokinesis and centrosome duplication. Mps1 predominantly resides in the cytoplasm and relocates into the nucleus at the late G2 phase. So far, the mechanism underlying the Mps1 translocation between the cytoplasm and nucleus has been unclear.

**Results:**

In this work, a dynamic export process of Mps1 from the nucleus to cytoplasm in interphase was revealed- a process blocked by the Crm1 inhibitor, Leptomycin B, suggesting that export of Mps1 is Crm1 dependent. Consistent with this speculation, a direct association between Mps1 and Crm1 was found. Furthermore, a putative nuclear export sequence (pNES) motif at the N-terminal of Mps1 was identified by analyzing the motif of Mps1. This motif shows a high sequence similarity to the classic NES, a fusion of this motif with EGFP results in dramatic exclusion of the fusion protein from the nucleus. Additionally, Mps1 mutant loss of pNES integrity was shown by replacing leucine with alanine which produced a diffused subcellular distribution, compared to the wild type protein which resides predominantly in cytoplasm.

**Conclusion:**

Taken these findings together, it was concluded that the pNES sequence is sufficient for the Mps1 export from nucleus during interphase.

**Electronic supplementary material:**

The online version of this article (doi:10.1186/s12860-015-0048-6) contains supplementary material, which is available to authorized users.

## Background

Mps1 (mono-polar spindles-1, also termed TTK in humans), a serine/threonine kinase, plays multiple functions in the cell cycle [1,2]. In mitosis, Mps1 is indispensable for both the spindle assembly checkpoint (SAC) and chromosomal alignment. Mps1 activates SAC by directing the assembly of the mitotic checkpoint complex (Cdc20, Mad2, Bub3 and BubR1). The resulting complex binds and inactivates APC/C-mediated proteolysis of Cyclin B and Securin [3,4]. Mps1 can phosphorylate Borealin to regulate chromosomal alignment independently of SAC [5,6]. Mps1 also affects centrosome duplication by collaborating with substrates, including Mortalin, Centrin 2 and VDAC3 [7-9]. In interphase, Mps1 interacts with p53 and Chk2 and is involved in DNA damage response [10-12].

The subcellular distribution of Mps1 correlates with the functions that it performs in the cell cycle. During mitosis, Mps1 appears on the kinetochore at prophase, stays consistently until metaphase initiation, and then comes off at metaphase upon mitotic entry [13-17]. Kinetochore association of Mps1 is regulated by Hec 1 and Aurora B [18]. The timely release of Mps1 from kinetochore is required for mitotic progression [19]. In interphase, Mps1 resides in the cytoplasm, nuclear envelope and centrosomes [14]. Translocation of Mps1 from cytoplasm to nucleus at the G2/M boundary requires two LXXLL motifs at the N-terminal [20]. The centrosome localization of Mps1 is required for centrosome duplication [21] and in the case of DNA damage, Mps1 is also recruited to DNA damage foci, showing that Mps1 plays a role in DNA damage response [10].

The regulatory mechanism retarding Mps1 at cytoplasm is unclear so far. In this paper, a putative nuclear export sequence (pNES) motif at the N-terminal of Mps1 was identified and this motif shows a high sequence similarity to the classic NES, exporting EGFP out of the nucleus in cases of fusion. Unexpectedly, replacing 1 isoleucine and 3 leucines in this pNES with alanines did not cause constitutive nuclear enrichment of Mps1. Further studies suggest that the 4 amino acid mutation affects Mps1 nuclear translocation at the G2/M boundary. Consistent with this finding, the Mps1 mutant with 1 of the last 2 leucines is predominantly cytoplasmic while the other mutants behave as with the 4 amino acid mutation. Collectively, the observations showed that the pNES sequence at the N-terminal is a functional motif which is sufficient for driving Mps1 nucleus export during interphase.

## Methods

### DNA constructs and stable cell lines generation

Retroviral expression vector pRex-YFP-Mps1-IRES-Hygro in the pRex background has been described previously [16]. Constructs pRex-EGFP-IRES-Hygro, pRK5-myc-Mps1, pEXL-FTH-Crm1, pGST-4 T-Mps1, pEXL-FTH-Crm1 were generated in a standard way. To generate pRex-pNESs-EGFP-IRES-hygro, the coding sequences for the putative NESs were achieved by annealing pairs of complementary oligo-nucleotide primers and then they were sub-cloned into the N-terminal of EGFP. To introduce the pNES1 mutations in Mps1, construct pRex-YFP-Mps1-IRES-Hygro was subject to a standard QuickChange mutagenesis procedure by PCR amplication with pairs of Oligos sequentially. Primers used in this paper are showed in Additional file 1: Table S1. All constructs were confirmed by DNA sequencing. The procedure for generation of SW480 stable cell lines expressing various Mps1 mutants is described.

### Protein expression and purification

To prepare protein from E.coli, the construct pQE60-Crm1 was transformed into BL21 (DE3) and then induced with 0.5 mM IPTG at 22°C overnight. The cell pellet was washed and suspended in binding buffer (0.5 mol/l NaCl, 0.1 mol/l Tris.Cl, pH8.0), followed by a regular sonication procedure. The cell debris was removed by centrifugation at 12,000 g for 30 min. The supernatant was then applied to Ni-charged chelating-Sepharose (Pharmacia Biotech, Uppsala, Sweden) and proteins were purified according to the protocol by the manufacturer. To produce the purified proteins from mammalian cells, plasmids were transciently transfected into 293 T cells. After 48 hrs, Cells were washed by D-PBS twice and then subject to lysis buffer (20 mM HEPES pH 7.6, 20% glycerol, 500 mM NaCl,1.5 mM MgCl2, 0.2 mM EDTA, 0.1% Triton X-100, 25 mM NaF, 25 mM glycerophosphate, 1 mM phenylmethylsulfonyl fluoride, 1 mM sodium orthovanadate, 1 mM dithiothreitol, 1× protease inhibitor).Then the supernatant was directly applied to Ni-charged chelating-Sepharose (Pharmacia Biotech, Uppsala, Sweden) or co-incubated with c-Myc Agarose Affinity beads for immunoprecipitation (Sigma-Aldrich) depending on the tag of proteins fused. Fusion proteins were quantified by BCA assay (Pierce, Rockford, USA) and the purity was confirmed by SDS-PAGE analysis.

### Cell synchronization

Synchronization of cells at G1/S phase was accomplished by sequential double thymidine and RO3306 treatment as described by Zhang *et al.* [11]. Briefly, cells were arrested at G1/S phase by treatment with 2 mM thymidine for 19 h and cells were washed 3 times with D-PBS before released into fresh DMEM medium for another 16 h. These cells were washed with D-PBS another 3 times and then released into DMEM medium with CDK1 inhibitor RO-3306 for 12 h. The arrested cells were then fixed with 1% paraformaldehyde for 15 min and subjected to immunofluorescence staining.

### Pulldown assay and Western blot analysis

To detect the direct interaction of proteins, equal amount purified proteins were co-incubated in buffer 1 (20 mM Tris-Cl (pH 8.0), 0.2 M NaCl, 0.5% NP40, 1 mM EDTA, 1 mM PMSF, 20 mM NaF, 0.1 mM Na3VO4, 1×Protease inhibitors(Roche)) and rotated for 2 h. Then the corresponding beads were added and rotated for another 1 h, the supernatant and beads were collected by centrifugation. The proteins in beads were released by boiling for 10 min in buffer 1. The proteins from supernatant and the boiled beads were resolved in 10% SDS-PAGE gel and transferred to nitrocellulose membrane. Primary and secondary antibodies were applied sequentially. Primary antibodies Anti-α GAPDH (Sigma), Anti-Mps1 NT (Abcam) and anti-6×His tag (Life technologies) were prepared at a 1:1000 dilution. The blots were developed in Super Signal WestDura (Pierce) according to the manufacturer’s instruction.

### Immunofluorescence

Cells for immunofluorescence were grown on cover glasses. Prior to staining, cells were treated with chemicals as indicated both in duration and doses. Cells were washed 3 times with D-PBS and fixed for 10 min in D-PBS plus 1% paraformaldehyde. Anti-γ-tubulin (Sigma) and Anti-Mps1 NT (Abcam) was prepared at a 1:1000 and 1:500 dilutions respectively. The cells were stained with primary antibody for 1.5 h at room temperature, followed by secondary antibodies conjugated with Alexa Fluor 488-conjugated goat anti-mice secondary antibodies (Invitrogen, Eugene, OR). To stain DNA, 50 μg/ml Propidium plus RNAase A or 1.5 μg/ml DAPI in D-PBS was used. After staining, the coverglasses were mounted onto pre-cleaned microscope slides with D-PBS containing 50% glycerol and sealed with nail oil. Images were acquired on a Zeiss LSM 510 equipped with a 63 × objective lens.

## Results

### Crm1 binds to and exports Mps1 from the nucleus after mitosis

**Figure 1:**
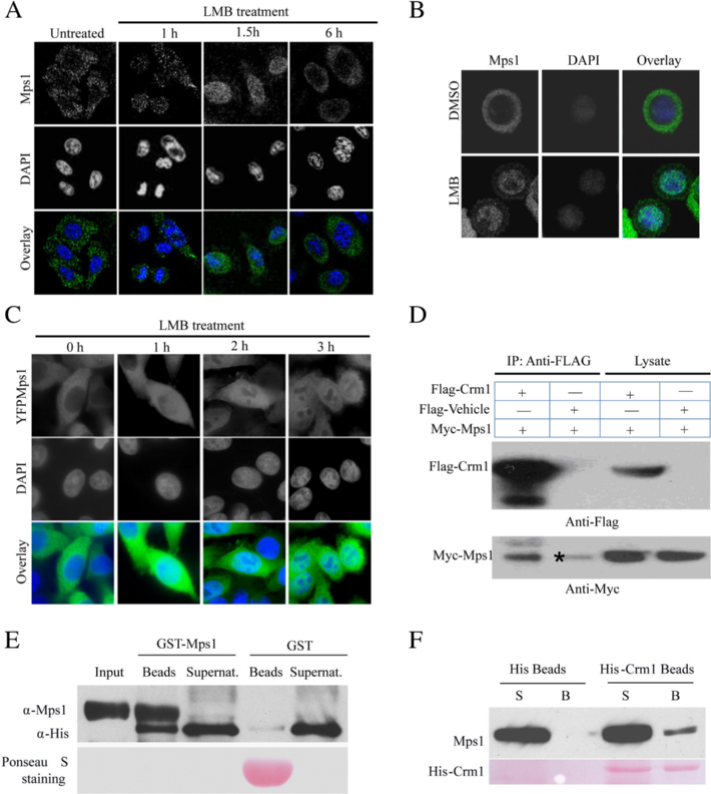
**Crm1 binds to and exports Mps1 from the nucleus after mitosis. (A)** Export of Mps1 from nucleus upon mitosis completion. SW480 cells were arrested at prometaphase via 100 ng/ml Nocodazole treatment and released into fresh medium for the duration indicated, before being fixed for immunofluorescence staining with anti-Mps1 N1 antibody. DNA was counterstained with DAPI. **(B)** Mps1 relocated into the nucleus upon LMB treatment. Asynchronized SW480 cells were treated with 10 μM LMB and fixed for immunofluorescence staining at the indicated time points. **(C)**YFP-fused Mps1 show same pattern in SW480 upon LMB treatment. Asynchronized SW480 cells expressing YFP-Mps1 were treated with 10 μM LMB and fixed for immunofluorescence staining at the indicated time points. **(D)** Interaction of Mps1 and Crm1. 293 T cells were transiently co-transfected with pEXL-FTH-Crm1 and pRK5-myc-Mps1 and collected for the immuno-precipitation assay. Nonspecific binding product is indicated as symbol *. **(E, F)** Reciprocal association of Mps1 and Crm1: The bead bound GST-Mps1 was co-incubated with His-crm1 for 3 hrs in lysis buffer and then was collected by centrifuge. The bead bound protein and the proteins in supernatant were examined by Western-blot with antibodies indicated. Reciprocally, bead bound His-Crm1 was incubated with a non-tagged Mps1. The interaction was also determined in the same standard protocol. DAPI, 4′,6-diamidino-2-phenylindole.

Mps1 dominantly resides in the cytoplasm and relocates into the nucleus at the G2/M boundary [20]. For the first time, it has been found that Mps1 can be excluded from the nucleus gradually as the nuclear envelope reforms after mitosis (Figure 1A). Cytoplasmic retardation of endogenous Mps1 in the colon cancer cell line SW480 requires Crm1, as treating cells with Crm1 inhibitor LMB can block Mps1 nuclear transport (Figure 1B). A similar result was also achieved by using stable SW480 cells expressing an YFP fused Mps1 (Figure 1C). These findings suggested that cytoplasmic retardation of Mps1 is due to the Crm1-mediated passive exclusion process. To determine that Crm1 directly regulates Mps1 translocation, the association of Mps1 with Crm1 was examined. 293 T cells were co-transfected with pEXL-FTH-Crm1 and pRK5-myc-Mps1 and collected for immuno-precipitation assay after 48 h. As shown in Figure 1D, Crm1 can bind to endogenous Mps1. To determine whether Mps1 can bind to Crm1 directly, an *in vitro* interaction of Mps1 and Crm1 was examined using purified proteins. GST-tagged Mps1 was purified from 293 T cells after a pFAST-GST-Mps1 baculovirus infection for 48 h. 6× His-tagged Crm1 was expressed and purified from *E. coli*. These 2 proteins were incubated with GST beads at room temperature for 2 h, followed by separating the supernatant and beads via centrifugation. Beads were washed 3× and the proteins on beads were subject to a western blot analysis. As showed in Figure 1E, a physical association of Mps1 with Crm1 was observed. A reciprocal interaction was also performed. As shown, His-crm1 can interact with a non-tag Mps1 (Figure 1F). Based on these findings, it was concluded that Crm1 binds Mps1 and may affect the nuclear export of Mps1 directly.

### Mps1 bears a putative nuclear export sequence

**Figure 2:**
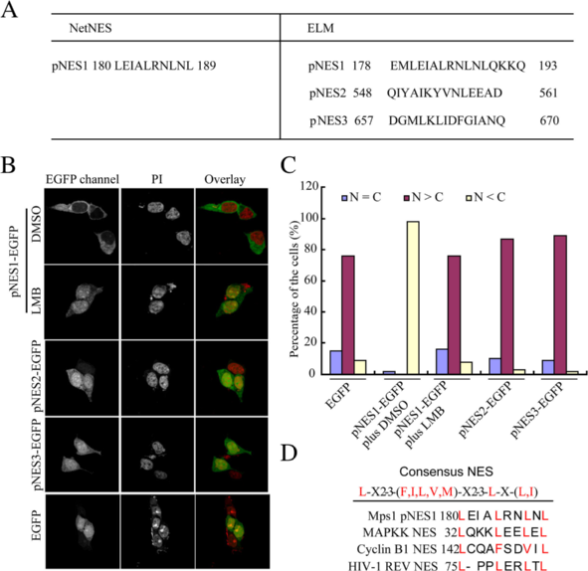
**Mps1 bears a putative nuclear export sequence. (A)** Prediction of nuclear export sequence in Mps1 via 2 online procedures. Mps1 protein sequence was loaded into procedures according to the protocol by software developer. The resulted nuclear sequences were showed as indicated. **(B, C)** Functional validation of the putative NES. The putative sequences were fused with EGFP in frame and the resulted constructs were transfected into 293 T cells. The subcellular distribution of fusion proteins were examined after 48 h under the conditions indicated. DNA was counterstained with PI. The ratio of fusion proteins in the nucleus and cytoplasm were measured via image J and the statistical result of these were generated by using Graphpad software. **(D)** The amino acids alignment of NES motifs. The pNES1 of Mps1 is aligned with the classical NES from MAPKK, Cyclin B1, HIV-REV. The letters in red indicate identical or similar amino acids. PI, Propidium Iodide.

Crm1 carries its cargo proteins through binding to a nuclear export sequence. Given the direct interaction of Mps1 and Crm1, it was speculated that Mps1 bears the nuclear export sequence(s). To uncover the putative NES, the Mps1 protein sequence was analyzed via 2 online software programs NetNES (*www.cbs.dtu.dk/services/NetNES/*) and ELM (*http://elm.eu.org/links.html*). As shown in Figure 2A, only 1 putative NES was revealed in both ELM and NetNES. To validate these prediction sequences, these putative NES sequences were fused to the N-terminal of enhanced-green fluorescence protein (EGFP) and the distribution of the resulted fusion protein was examined after a transient transfection of 293 T cells. Only the pNES1 fused EGFP resided in the cytoplasm while the other 2 pNES fusion proteins showed a similar distribution pattern as that of the EGFP. The cytoplasmic distribution of pNES1-EGFP appears to rely on Crm1, as the treatment of cells with LMB caused the nuclear enrichment of pNES1-fused EGFP (Figure 2B and C). The pNES1 seemed to be a classic NES sequence as the leucines enriched pattern is conserved when aligned to the classic NES sequence of Cyclin B1, HIV Rev and MAPK (Figure 2D). Notably, replacement of these conserved leucines with alanine caused re-localization of EGFP into the nucleus (Additional file 2: Figure S1). Based on these findings, it is thought that pNES1 in Mps1 is a functional NES.

### Mps1 with all leucines substituted in pNES1 reside in the cytoplasm

**Figure 3:**
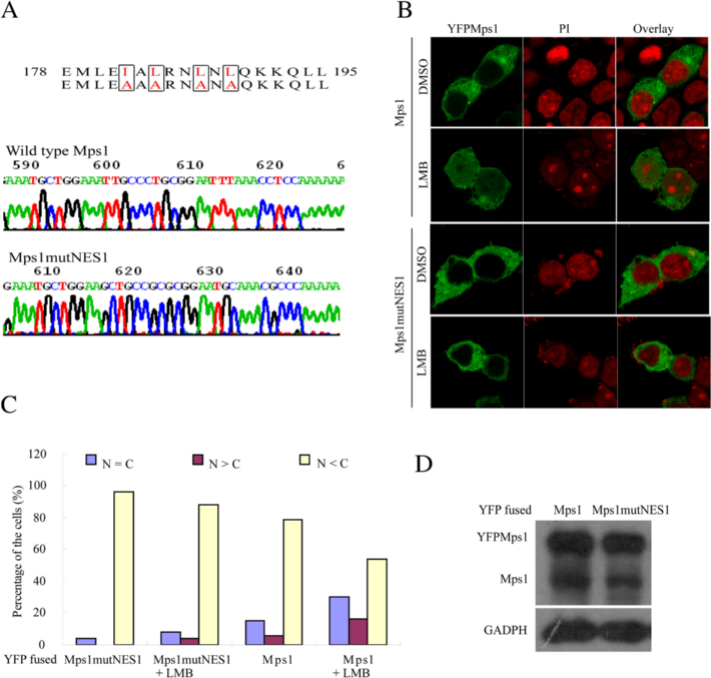
**Mps1 with all leucines substituted in pNES1 reside in the cytoplasm. (A)** Schematic of Mps1 mutant with mutated pNES1 (Mps1mutNES1). The lysines in red were mutated to alanines (upper panel). The sequencing result of the mutated pNES1 (middle and lower panel). **(B, C)** The subcellular distribution of YFP fused Mps1 and Mps1mutNES1 in presence of LMB and DMSO. The quantitative result of fusion distribution was generated as in Figure 2C. **(D)** Western blot of 293 T cells shows the expression of YFPMps1 and YFPMps1mutNES1 in SW480 cell lines.

To further validate that pNES1 is a functional NES, The subcellular distribution of Mps1 mutants, in which 1 isoleucine and 3 leucines were replaced with alanines (Mps1mutNES1) was determined. The resulted fusion protein YFPMps1mutNES1 was forcibly expressed in 293 T cells and the subcellular distribution was examined under the indications as shown (Figure 3A, D). Unexpectedly, the Mps1mutNES1 did not reside in the nucleus, even in the presence of LMB (Figure 3B and C), suggesting that pNES1 may be not sufficient for nuclear export, or that this mutation may affect both the nuclear import and export of Mps1 in interphase.

### Mps1 with one leucine substituted in pNES1 relocate to the nucleus

**Figure 4:**
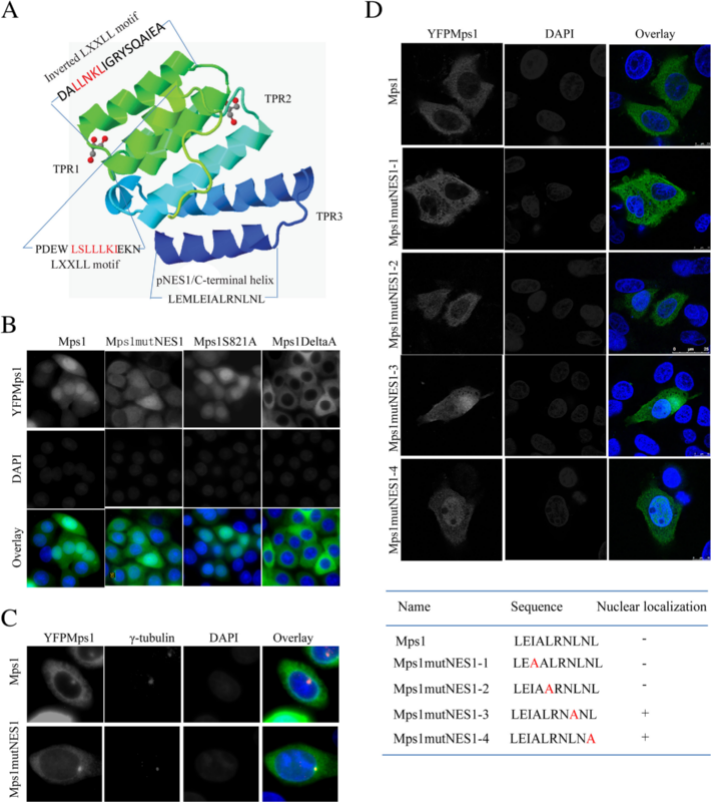
**Mps1 with a single leucine substituted in pNES1 relocate to the nucleus. (A)** pNES1 motif is adjacent to the LXXLL motifs within TPR domain. **(B)** The subcellular distribution of wild type Mps1, Mps1 S821A, Mps1mutNES1, Mps1deltaA domain at G2/M boundary. **(C)** The centrosome localization of wild type Mps1 and Mps1mutNES. **(D)** The subcellular distribution of wild type Mps1, Mps1mutNES1-1, Mps1mutNES1-2, Mps1mutNES1-3, Mps1mutNES1-4 in interphase.

Mps1 moves from the cytosol to the nucleus at the G2/M boundary prior to the nuclear envelope breakdown (NEB) and this process depends on 2 LXXLL motifs which contribute to the partial Tetratricopeptide repeat domain (TPR) in the N-terminus of Mps1[20,22]. Given that the pNES1 is adjacent to the TPR domain (Figure 4A), it was proposed that the mutation of pNES1 may alter the conformation of these peptides adjacent to the LXXLL motifs. In this case, the Mps1mutpNES1 would fail to get into the nucleus at the G2/M boundary. To test this theory, it was observed that the distribution of YFP fused Mps1 and Mps1mutNES1 was expressed constantly in SW480 and was arrested at the G2 phase by sequentially treating cells with thymidine and CDK1 inhibitor RO-3306. Consistent with the previous report [20], the Mps1 mutant loss of 2 LXXLL motifs in the A domain (Mps1deltaA) failed to get into the nucleus at late G2 phase as compared with Mps1. As a control, the nonphosphorylation mimics of Mps1 at S821 (Mps1S821A), which is phosphorylated by MAP kinase, was translocated into the nucleus at the G2/M boundary [23]. Interestingly, Mps1mutNES1 showed an impaired nuclear localization (Figure 4B), showing that all leucine substitutions affect the nuclear localization of Mps1. In contrast, Mps1mutNES1 localized to the centrosome as efficiently as wild type Mps1 (Figure 4C), suggesting the nuclear entry inefficiency is not due to the misfolding of the N-terminal. To examine the amino acids essential for Mps1 nuclear export, YFP fused Mps1 mutants with only 1 mutated leucine were generated and evaluated for the efficiency of nuclear entry. Notably, these mutants show the diverse localization pattern: mutation 1 of the forward isoleucine (Mps1mutNES1-1) or leucine (Mps1mutNES1-2) predominantly resides in cytoplasm while the mutants with 1 of the last 2 leucines replaced (Mps1mutNES1-3 and (Mps1mutNES1-4) show an apparent nuclear localization (Figure 4D). Similar result was obtained when SW480 cells expressing these mutants were arrested at the late G2 phase by using CDK1 inhibitor RO-3306 (Additional file 3: Figure S2). These findings suggest the pNES1 is a functional nuclear export sequence and mutation of the pNES1 sequences affect the overall structure integrity of Mps1 and thereby the distribution in interphase.

## Discussion and conclusions

Mps1 predominantly resides in cytoplasm during interphase and then translocates into the nucleus at the G2/M boundary [20]. These observations reveal another Mps1 translocation process in interphase. Upon completion of mitosis, Mps1 was gradually excluded from the nucleus, mediated by Mps1/Crm1 interaction. Furthermore, a putative NES motif at the N-terminal of Mps1 was identified. Fusion of this motif with EGFP causes the latter to be restrained in the cytoplasm. In addition, it was found that substitution of 1 leucine is sufficient to relocate the Mps1 into the nucleus although the complete substitution of leucine actually hampers this effect. This NES, however, seems to be not the only mediator for Mps1 export after mitosis as not all Mps1 accumulate in the nucleus, suggesting that another layer of regulation mechanisms is also involved in this process. TP53 is an attractive candidate- this can bind Mps1 in cases of DNA damage response [10]. Nuclear export of TP53 is mediated by MDM2 which bears a classic NES [24]. As a result, a straightforward hypothesis is that Mps1 may be exported into cytoplasm by interacting with other proteins, such as TP53.

The N-terminal of Mps1 is essential several versatile roles, including kinetochore localization, protein instability and nuclear translocation [17,20,25]. Recently, the crystal structure of the Mps1 N-terminus was solved independently by 2 groups [22,26]. The Mps1 N-terminal is characterized with a tetratricopeptide repeat (TPR) domain, followed by the pNES1. Due to LXXLL motifs being protein-protein interaction domains but not classic nuclear localization sequences [27], it is speculated that the disruption of pNES1 may affect the nuclear import of Mps1 in interphase. Consistent with this hypothesis, the nuclear translocation of Mps1mutNES1 is impaired at the G2/M boundary, showing that the disruption of pNES1 integrity also affects Mps1 nuclear translocation. It is noted that the pNES1 sequence of Mps1 is not orthologically conserved among human, mouse, frog and zebrafish (Additional file 4: Figure S3).It is speculated that beside pNES1, other unidentified sequence elements are involved in cytoplasmic restraint of Mps1 in interphase.

The results reveal that Mps1 is a novel target for Crm1. Nevertheless, the function underlying the association of Mps1 with Crm1 has been unclear so far. As well as Mps1, Crm1 also regulates the subcellular distribution of several other mitotic regulators in interphase, including Cyclin B, Aurora B and Aurora A [28,29]. These substrates are crucial for several aspects of mitosis, including mitotic spindle formation, nuclear envelope breakdown, chromosomal alignment and SAC generation. Crm1 is also an active mitotic regulator: including the recruitment of Mad1 to the kinetochore, promotion of spindle assembly and regulation of mitotic progression and chromosome segregation [30-32]. Recently, it was found that Crm1 may also regulate mitotic progression via Mps1 as LMB treatment can promote kinetochore recruitment of Mps1 through mitosis (unpublished data). Collectively, the results here add new regulatory roles of Crm1 in Mps1 translocation during interphase.

## Additional files

Additional file 1: Table S1. The primers used in this paper. Additional file 2: Figure S1. Plasmids bearing pNES1-EGFP mutant as indicated were transfected to SW480 and imaged for the localization of fusion protein after 48 hours. Additional file 3: Figure S2. Subcellular distribution of YFP-Mps1 pNES1 mutants in SW480 cells arrested at the late G2 phase. Additional file 4: Figure S3. The alignment of Mps1 orthologue from mouse, human, frog and zerafish. The alignment process was conducted by DNAssist software and presented by Photoshop software.

## Abbreviations

Mps1: Mono-Polar Spindles-1
pNES: Putative nuclear export sequence
SAC: Spindle Assembly Checkpoint
NEB: Nuclear Envelope Breakdown
TPR: Tetratricopeptide repeat domain
APC/C: Anaphase Promoting Complex/cyclosome
Crm1: Chromosome region maintenance 1
VDAC3: Voltage-dependent anion-selective channel protein 3
MARK: Microtubule affinity-regulating kinase
LMB: Leptomycin B
DAPI: 4, 6-diamidino-2-phenylindole
EGFP: Enhanced Green Fluorescent Portein
YFP: Yellow Fluorescent Protein
HIV: Human Immunodeficiency Virus
CDK1: Cyclin-dependent kinase 1
GST: Glutathione S Transferase
GAPDH: Glyceraldehyde-3-phosphate dehydrogenase
SDS-PAGE: Sodium dodecyl sulfate-polyacrylamide gelelectrophoresis
