# Supplementary figures and images for: A putative N-terminal nuclear export sequence is sufficient for Mps1 nuclear exclusion during interphase
Source: BMC Cell Biol. 2015 Feb 27;16:6. doi: 10.1186/s12860-015-0048-6 (PMC4373099; doi:10.1186/s12860-015-0048-6)

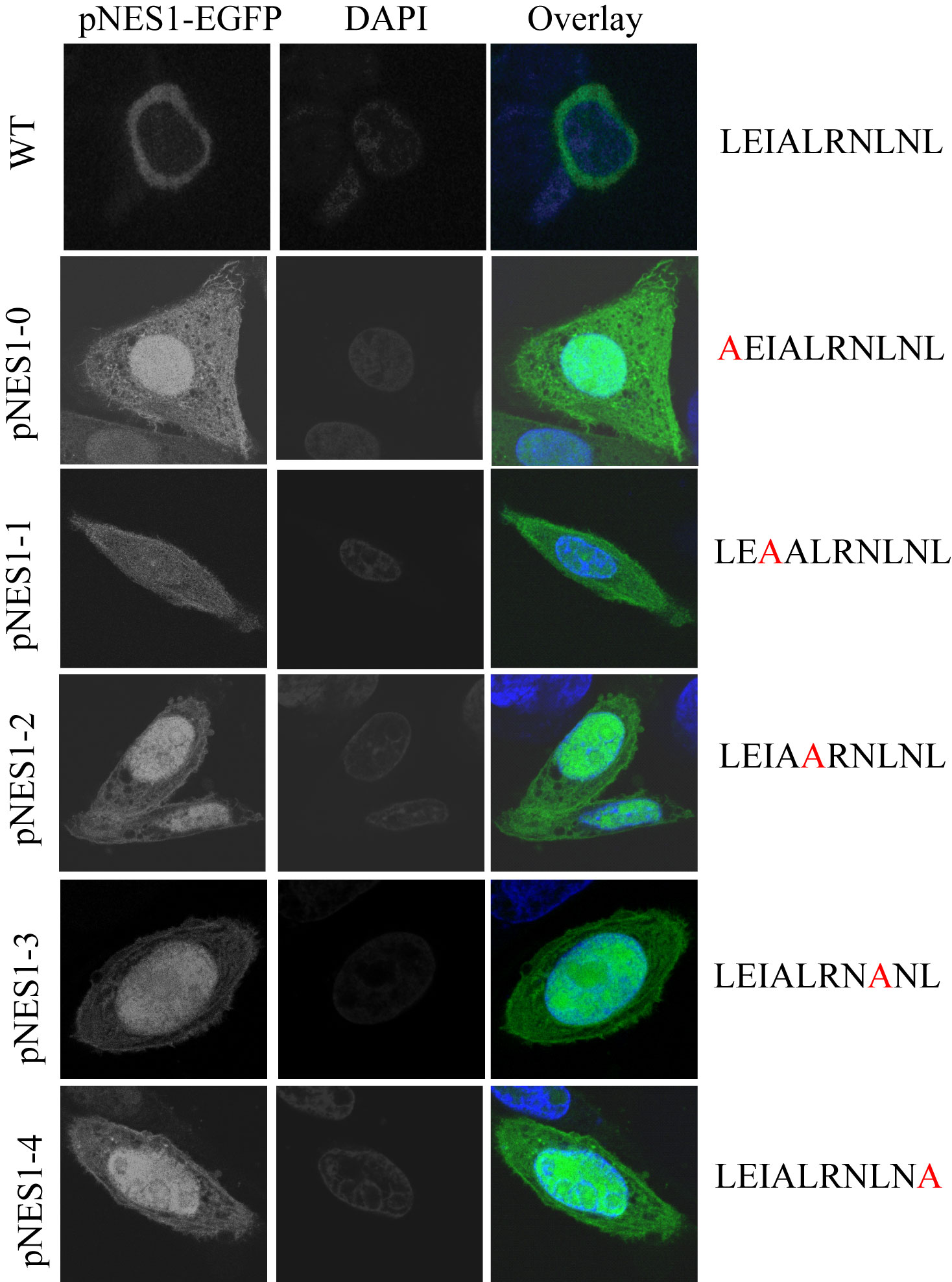

Supplement: Additional file 2: Figure S1. — Plasmids bearing pNES1-EGFP mutant as indicated were transfected to SW480 and imaged for the localization of fusion protein after 48 hours. [file 12860_2015_48_MOESM2_ESM.jpeg]

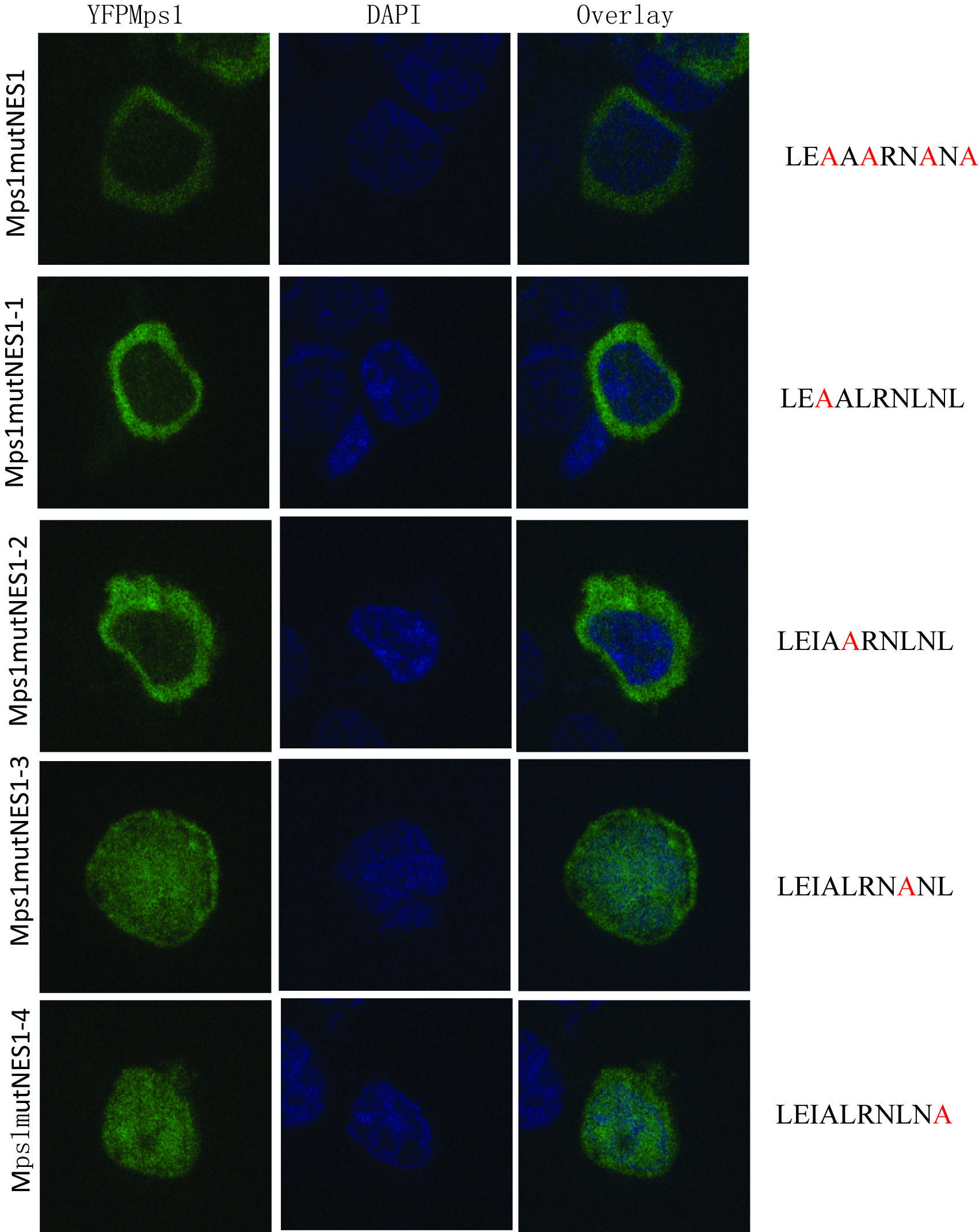

Supplement: Additional file 3: Figure S2. — Subcellular distribution of YFP-Mps1 pNES1 mutants in SW480 cells arrested at the late G2 phase. [file 12860_2015_48_MOESM3_ESM.jpeg]

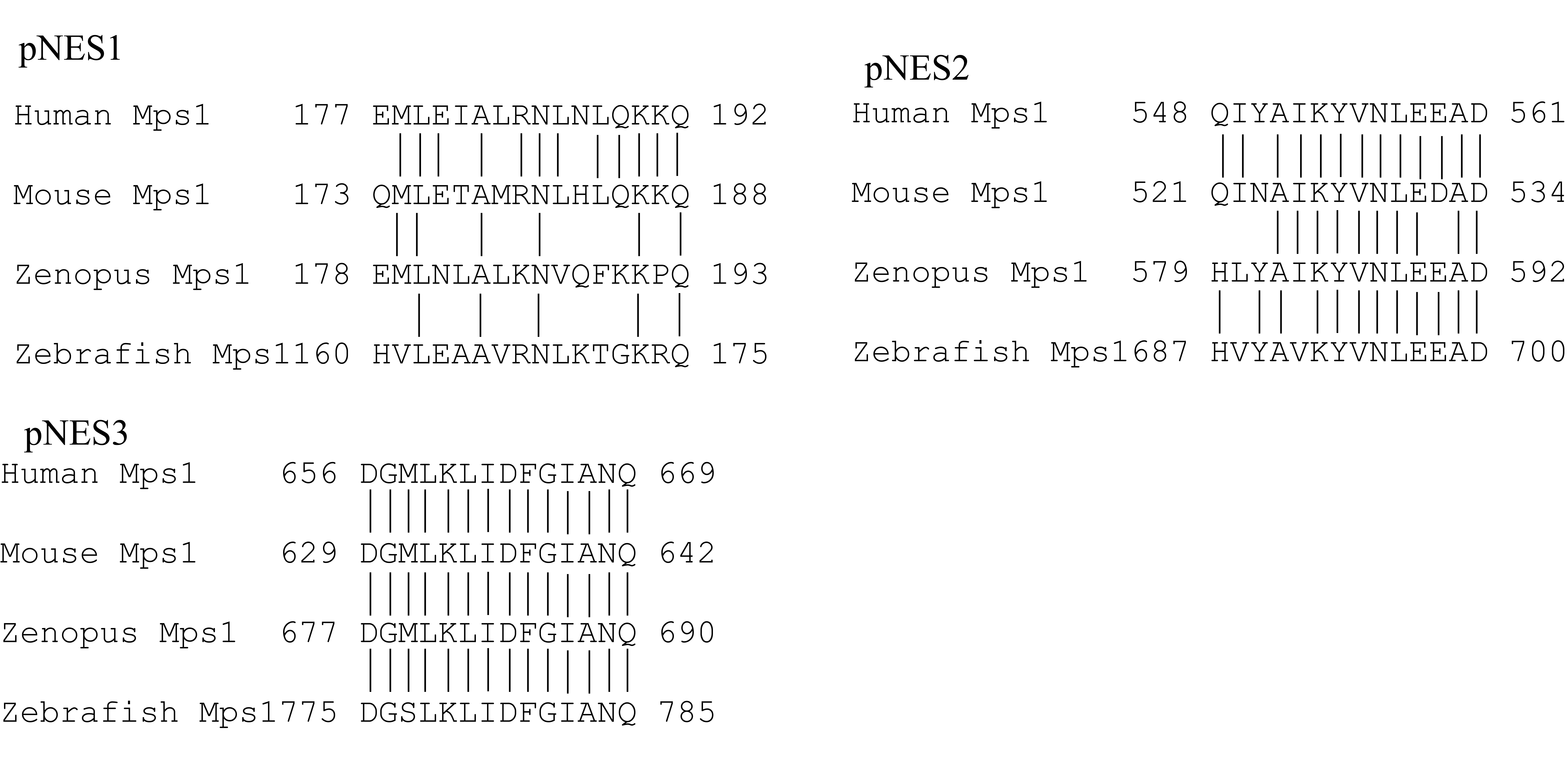

Supplement: Additional file 4: Figure S3. — The alignment of Mps1 orthologue from mouse, human, frog and zerafish. The alignment process was conducted by DNAssist software and presented by Photoshop software. [file 12860_2015_48_MOESM4_ESM.jpeg]
